# Porphyryne

**DOI:** 10.1021/acsomega.2c05199

**Published:** 2022-10-25

**Authors:** Abhik Ghosh, Jeanet Conradie

**Affiliations:** † Department of Chemistry, UiT − The Arctic University of Norway, N-9037 Tromsø, Norway; ‡ Department of Chemistry, University of the Free State, P.O. Box 339, Bloemfontein 9300, South Africa

## Abstract

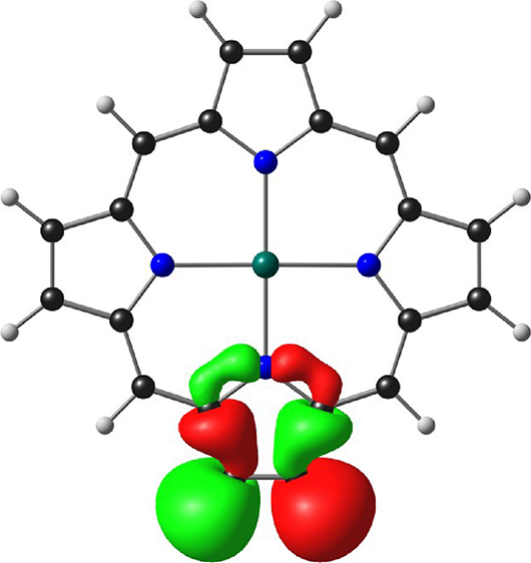

Density functional theory calculations with the B3LYP*-D3 method with large STO-QZ4P basis sets unambiguously predict a singlet ground state for Zn-porphyryne. However, the calculations also predict a low singlet–triplet gap of about 0.4 eV and a high adiabatic electron affinity of 2.4 eV. Accordingly, the reactivity of porphyryne species may be dominated by electron transfer, hydrogen abstraction, and proton-coupled electron transfer processes.

## Introduction

Although the existence of benzyne (1,2-dehydrobenzene) was surmised in the early part of the 20th century,^1^ strong evidence for the species emerged decades later, most notably through the work of Wittig^2−5^ and Roberts.^6−11^ Subsequently, benzyne has been intensively studied via gas-phase spectroscopic studies, especially photoelectron spectroscopy,^12−14^ and quantum chemical calculations.^15−18^ More recently, benzyne has been trapped in a container molecule^19^ and even imaged with STM.^20^ As powerful Diels–Alder dienophiles, benzynes and other arynes are widely employed as highly reactive synthetic reagents and intermediates.^21−24^ Benzynes are key intermediates of the hexadehydro-Diels–Alder reaction, a synthetic reaction recently developed by Hoye and coworkers.^25,26^ Interestingly, in spite of major advances in the functionalization of porphyrin-type compounds,^27−29^ an aryne based on a porphyrin, i.e., a porphyryne, remains unknown. Herein, following a long-standing tradition among chemical theoreticians in studying reasonable-looking but nonexistent molecules,^30−35^ we have considered the viability of such an intermediate based on a comparative density functional theory (DFT) study of zinc porphyryne and benzyne.

## Results and Discussion

**Figure 1:**
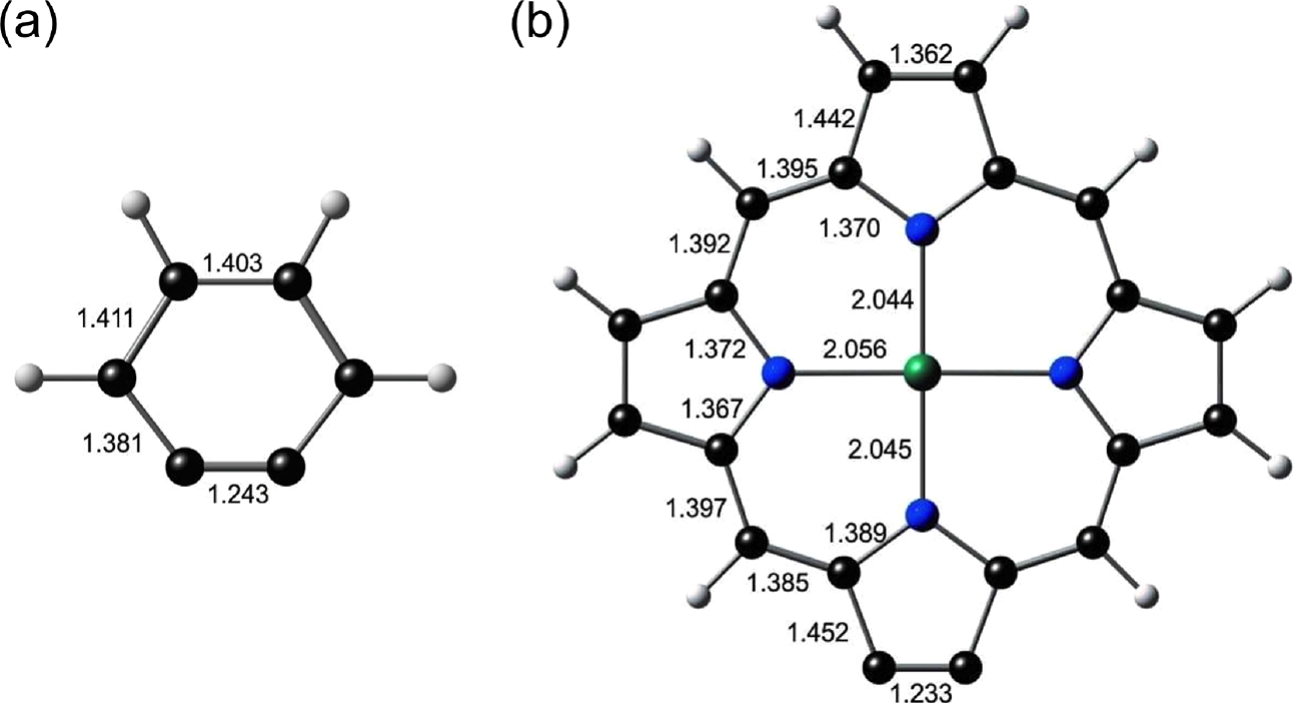
Symmetry-distinct bond distances (Å) in all-electron B3LYP*-D3/ZORA-STO-QZ4P-optimized geometries of (a) benzyne and (b) Zn-porphyryne. Note that a *C*_2*v*_ minimum was obtained for each molecule. Note also very similar C–C triple-bond distances in the two molecules.

**Figure 2:**
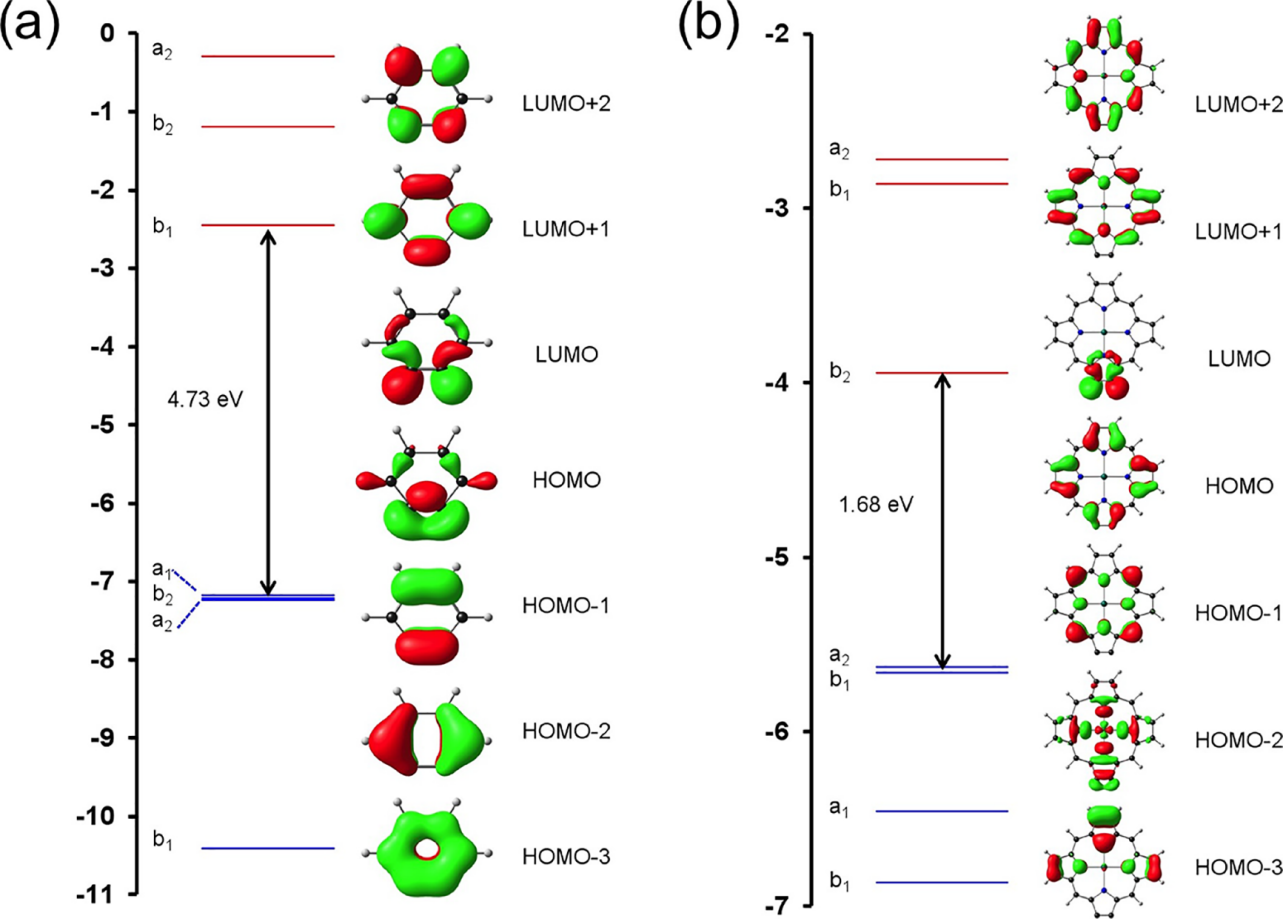
B3LYP*-D3/ZORA-STO-QZ4P Kohn–Sham MO energy level diagrams for (a) benzyne and (b) Zn-porphyryne.

**Figure 3:**
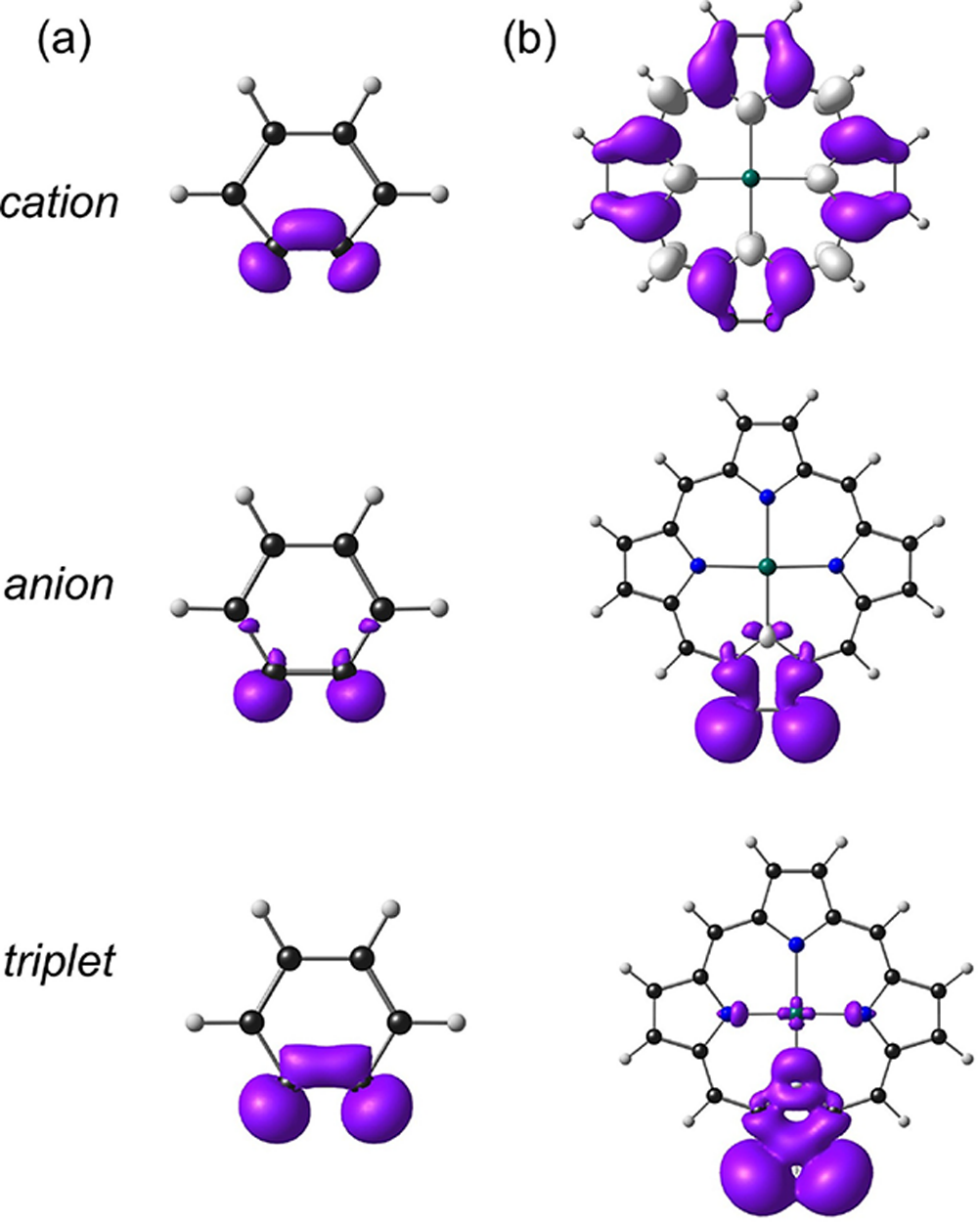
B3LYP*-D3/ZORA-STO-QZ4P spin density profiles (0.002 e·Å^–3^) of selected adiabatically ionized/excited states of (a) benzyne and (b) Zn-porphyryne.

While a variety of popular exchange-correlation functionals were examined (which generally yielded very similar results), the results quoted (Table 1 and Figures 1–3) are those for the well-tested^36−40^ hybrid functional B3LYP* (with 15% Hartree–Fock exchange)^41,42^ augmented with Grimme’s D3 dispersion corrections^43^ as implemented in the ADF 2019 program system.^44,45^ The optimized geometry of Zn-porphyryne corresponds to *C*_2*v*_ symmetry and reveals unremarkable skeletal bond distances, except for that of the C–C triple bond (1.233 Å), which is similar to that in benzyne (1.243 Å; Figure 1). Kohn–Sham MO energy level diagrams facilitate further comparative discussion of the two molecules (Figure 2). For benzyne, although the HOMO corresponds to the in-plane triple-bond π-MO, it is accidentally degenerate with two other benzene π-HOMOs. Thus, our calculations predict three near-degenerate IPs, an interesting facet of benzyne that appears to have been overlooked in the literature until now (Table 1).^12−18^ In the case of Zn-porphyryne, the two HOMOs correspond to the classic Gouterman a_1u_ and a_2u_ HOMOs,^46−49^ while the in-plane triple-bond π-MO corresponds to HOMO-2. Accordingly, the vertical IP corresponding to ionization from the latter MO (7.9 eV) is about an eV higher than the two lowest IPs (6.9 eV, which is essentially the same as that calculated for Zn-porphyrin and experimentally observed for unsubstituted free-base porphine^50^).^51−55^ For both benzyne and Zn-porphyryne, the LUMO clearly corresponds to the in-plane triple-bond π-antibonding MO. Another point of similarity is that the triplet state of both molecules involves an excitation from the in-plane triple-bond π-MO to the triple-bond π*-MO and is thus characterized by similar spin density profiles.

**Table 1:** Scalar-Relativistic All-Electron B3LYP*-D3/ZORA-STO-QZ4P Results on Benzyne, Zn-Porphyryne, and Zn-Porphyrin: IPs, EAs, and Singlet–Triplet Gaps (eV)

	vertical			adiabatic				
compound	IP1	IP2	IP3	EA	*E*_S–T_	IP1	IP2	IP3
benzyne	9.610	9.637	9.616	0.645	1.457	9.480	9.481	9.483
Zn-porphyryne	6.928	6.963	7.936	2.397	0.441	6.886	6.939	7.790
Zn-porphyrin^a^	6.759	6.764	7.915	1.201	1.830	6.691		

a The adiabatic IP and EA of Zn-porphyrin take into account their different point group symmetries relative to the neutral state, as a result of Jahn–Teller-type symmetry-breaking phenomena in the ionized states.

Our calculations also underscore major differences between benzyne and Zn-porphyryne as well as between Zn-porphyryne and Zn-porphyrin. Thus, Zn-porphyryne is expected to exhibit a high electron affinity (2.4 eV) and a low singlet–triplet gap (0.44 eV). For reference, a simple closed-shell porphyrin such as Zn-porphyrin (Table 1) exhibits a much lower electron affinity^56−60^ of around 1.2 eV and a much higher singlet–triplet gap of ∼2.0 eV.^61^ Both features of Zn-porphyryne appear to be a consequence of the low LUMO energy level (well below the Gouterman π-LUMOs^46,47^) and the lower HOMO–LUMO gap relative to benzyne, which in turn presumably reflect the greater destabilization of a triple bond in a five-membered ring relative to a six-membered ring (in spite of similar triple-bond distances, as shown in Figure 1).

## Conclusions

High-quality DFT calculations clearly indicate Zn-porphyryne as a ground-state singlet species. Its high electron affinity, however, may thwart classic reaction pathways such as the Diels–Alder reaction. Instead, major reaction pathways may involve electron transfer, C–H abstraction, and proton-coupled electron transfer. Nevertheless, the overall calculated energetics suggest the existence of porphyryne species as reactive intermediates, and we accordingly encourage experimentalists to attempt their generation and trapping under carefully controlled conditions.

## Supporting Information Available

Optimized Cartesian coordinates (PDF)
