# Supplementary material for: Porphyryne
Source: ACS Omega. 2022 Oct 25;7(44):40275–8. doi: 10.1021/acsomega.2c05199 (PMC9647813; doi:10.1021/acsomega.2c05199)
Supplement: Supplementary file 1 — ao2c05199_si_001.pdf [file ao2c05199_si_001.pdf]

Supporting Information

Porphyryne

Abhik Ghosh[a,*] and Jeanet Conradie[a,b,*]

[a] Department of Chemistry, UiT – The Arctic University of Norway, N-9037 Tromsø, Norway;

Email: abhik.ghosh@uit.no

[b] Department of Chemistry, University of the Free State, P.O. Box 339, Bloemfontein, 9300, South Africa

B3LYP*-D3/ZORA-STO-QZ4P optimized Cartesian coordinates (Å)

1. Benzyne 2
2. Zn-porphyryne, C_{2v}, q = 0, S = 0 2
3. Zn-porphyryne, C_{2v}, q = 0, S = 1 3
4. Zn-porphyryne, C_{2v}, q = +1, S = ½, adiabatic cationic state 1 . 3
5. Zn-porphyryne, C_{2v}, q = +1, S = ½, adiabatic cationic state 2 . 4
6. Zn-porphyryne, C_{2v}, q = +1, S = ½, adiabatic cationic state 3 . 5
7. Zn-porphyryne, C_{2v}, q = -1, S = ½, adiabatic anion 6
8. Zn-porphyrin, D_{4h}, q = 0, S = 0 6
9. Zn-porphyrin, C_1, q = 0, S = 1 7
10. Zn-porphyrin, C_{4h}, q = +1, S = ½, adiabatic cationic state 1 .. 8
11. Zn-porphyrin, D_{2h}, q = -1, S = ½, adiabatic anion 9

1. Benzyne

C	0.621556000	0.000000000	-1.170673000
C	-0.621556000	0.000000000	-1.170673000
C	1.458492000	0.000000000	-0.072610000
C	-1.458492000	0.000000000	-0.072610000
C	0.701323000	0.000000000	1.117597000
C	-0.701323000	0.000000000	1.117597000
H	2.539371000	0.000000000	-0.073042000
H	-2.539371000	0.000000000	-0.073042000
H	1.225582000	0.000000000	2.066881000
H	-1.225582000	0.000000000	2.066881000

2. Zn-porphyryne, C_{2v}, q = 0, S = 0

Zn	0.000000000	0.000000000	-0.023187000
N	0.000000000	2.055811000	-0.031236000
N	0.000000000	0.000000000	-2.068175000
N	0.000000000	-2.055811000	-0.031236000
N	0.000000000	0.000000000	2.020825000
C	0.000000000	4.251276000	-0.706488000
C	0.000000000	2.871116000	-1.128897000
C	0.000000000	1.120905000	-2.888409000
C	0.000000000	0.616713000	-4.250452000
C	0.000000000	2.437215000	-2.457247000
C	0.000000000	2.868739000	1.074351000
C	0.000000000	4.249477000	0.654410000
C	0.000000000	-0.616713000	-4.250452000
C	0.000000000	-1.120905000	-2.888409000
C	0.000000000	-2.437215000	-2.457247000
C	0.000000000	-2.871116000	-1.128897000
C	0.000000000	-4.251276000	-0.706488000
C	0.000000000	-4.249477000	0.654410000
C	0.000000000	-2.868739000	1.074351000
C	0.000000000	-1.102243000	2.834370000
C	0.000000000	-0.680795000	4.213844000
C	0.000000000	0.680795000	4.213844000
C	0.000000000	1.102243000	2.834370000
C	0.000000000	2.425531000	2.393680000
C	0.000000000	-2.425531000	2.393680000
H	0.000000000	-1.345372000	5.064365000
H	0.000000000	1.345372000	5.064365000
H	0.000000000	5.099332000	1.319956000
H	0.000000000	5.102151000	-1.370596000
H	0.000000000	-5.102151000	-1.370596000
H	0.000000000	-5.099332000	1.319956000
H	0.000000000	3.200287000	-3.223734000
H	0.000000000	-3.200287000	-3.223734000
H	0.000000000	-3.190119000	3.161090000

H	0.000000000	3.190119000	3.161090000

3. Zn-porphyryne, C_{2v}, q = 0, *S* = 1

Zn	0.000000000	0.000000000	-0.019794000
N	0.000000000	2.039889000	-0.032950000
N	0.000000000	0.000000000	-2.100437000
N	0.000000000	-2.039889000	-0.032950000
N	0.000000000	0.000000000	2.030344000
C	0.000000000	4.239411000	-0.703927000
C	0.000000000	2.862258000	-1.131877000
C	0.000000000	1.122677000	-2.905301000
C	0.000000000	0.678765000	-4.268625000
C	0.000000000	2.437412000	-2.456877000
C	0.000000000	2.852180000	1.073917000
C	0.000000000	4.233049000	0.655988000
C	0.000000000	-0.678765000	-4.268625000
C	0.000000000	-1.122677000	-2.905301000
C	0.000000000	-2.437412000	-2.456877000
C	0.000000000	-2.862258000	-1.131877000
C	0.000000000	-4.239411000	-0.703927000
C	0.000000000	-4.233049000	0.655988000
C	0.000000000	-2.852180000	1.073917000
C	0.000000000	-1.101544000	2.843786000
C	0.000000000	-0.680282000	4.224572000
C	0.000000000	0.680282000	4.224572000
C	0.000000000	1.101544000	2.843786000
C	0.000000000	2.420188000	2.397802000
C	0.000000000	-2.420188000	2.397802000
H	0.000000000	-1.345318000	5.074739000
H	0.000000000	1.345318000	5.074739000
H	0.000000000	5.079748000	1.325418000
H	0.000000000	5.091533000	-1.366332000
H	0.000000000	-5.091533000	-1.366332000
H	0.000000000	-5.079748000	1.325418000
H	0.000000000	3.205668000	-3.219551000
H	0.000000000	-3.205668000	-3.219551000
H	0.000000000	-3.191623000	3.157898000
H	0.000000000	3.191623000	3.157898000

4. Zn-porphyryne, C_{2v}, q = +1, *S* = ½, adiabatic cationic state 1

Zn	0.000000000	0.000000000	-0.025364000
N	0.000000000	2.047771000	-0.031760000
N	0.000000000	0.000000000	-2.059154000
N	0.000000000	-2.047771000	-0.031760000
N	0.000000000	0.000000000	2.010814000
C	0.000000000	4.259782000	-0.701028000
C	0.000000000	2.862887000	-1.126336000

C	0.000000000	1.117425000	-2.881411000
C	0.000000000	0.614194000	-4.252375000
C	0.000000000	2.436311000	-2.457069000
C	0.000000000	2.861085000	1.070988000
C	0.000000000	4.258180000	0.648411000
C	0.000000000	-0.614194000	-4.252375000
C	0.000000000	-1.117425000	-2.881411000
C	0.000000000	-2.436311000	-2.457069000
C	0.000000000	-2.862887000	-1.126336000
C	0.000000000	-4.259782000	-0.701028000
C	0.000000000	-4.258180000	0.648411000
C	0.000000000	-2.861085000	1.070988000
C	0.000000000	-1.099269000	2.824618000
C	0.000000000	-0.674829000	4.220965000
C	0.000000000	0.674829000	4.220965000
C	0.000000000	1.099269000	2.824618000
C	0.000000000	2.425142000	2.392627000
C	0.000000000	-2.425142000	2.392627000
H	0.000000000	-1.342983000	5.068161000
H	0.000000000	1.342983000	5.068161000
H	0.000000000	5.104603000	1.317578000
H	0.000000000	5.107116000	-1.368975000
H	0.000000000	-5.107116000	-1.368975000
H	0.000000000	-5.104603000	1.317578000
H	0.000000000	3.198445000	-3.223571000
H	0.000000000	-3.198445000	-3.223571000
H	0.000000000	-3.188530000	3.159765000
H	0.000000000	3.188530000	3.159765000

5. Zn-porphyryne, C_{2v}, q = +1, S = ½, adiabatic cationic state 2

Zn	0.000000000	0.000000000	-0.019776000
N	0.000000000	2.057910000	-0.030931000
N	0.000000000	0.000000000	-2.075531000
N	0.000000000	-2.057910000	-0.030931000
N	0.000000000	0.000000000	2.025563000
C	0.000000000	4.255657000	-0.708701000
C	0.000000000	2.877348000	-1.124574000
C	0.000000000	1.113145000	-2.900722000
C	0.000000000	0.617345000	-4.255038000
C	0.000000000	2.434346000	-2.454431000
C	0.000000000	2.873926000	1.066735000
C	0.000000000	4.253410000	0.654698000
C	0.000000000	-0.617345000	-4.255038000
C	0.000000000	-1.113145000	-2.900722000
C	0.000000000	-2.434346000	-2.454431000
C	0.000000000	-2.877348000	-1.124574000
C	0.000000000	-4.255657000	-0.708701000
C	0.000000000	-4.253410000	0.654698000
C	0.000000000	-2.873926000	1.066735000

C	0.000000000	-1.096402000	2.842383000
C	0.000000000	-0.681813000	4.220863000
C	0.000000000	0.681813000	4.220863000
C	0.000000000	1.096402000	2.842383000
C	0.000000000	2.422152000	2.391702000
C	0.000000000	-2.422152000	2.391702000
H	0.000000000	-1.345076000	5.071818000
H	0.000000000	1.345076000	5.071818000
H	0.000000000	5.103310000	1.319313000
H	0.000000000	5.107261000	-1.371114000
H	0.000000000	-5.107261000	-1.371114000
H	0.000000000	-5.103310000	1.319313000
H	0.000000000	3.197925000	-3.221615000
H	0.000000000	-3.197925000	-3.221615000
H	0.000000000	-3.188026000	3.158225000
H	0.000000000	3.188026000	3.158225000

6. Zn-porphyryne, C_{2v}, q = +1, S = ½, adiabatic cationic state 3

Zn	0.000000000	0.000000000	-0.004593000
N	0.000000000	2.014914000	-0.044653000
N	0.000000000	0.000000000	-2.086589000
N	0.000000000	-2.014914000	-0.044653000
N	0.000000000	0.000000000	2.008552000
C	0.000000000	4.219763000	-0.698248000
C	0.000000000	2.854065000	-1.137310000
C	0.000000000	1.153979000	-2.893372000
C	0.000000000	0.621156000	-4.212366000
C	0.000000000	2.458458000	-2.463787000
C	0.000000000	2.827116000	1.072800000
C	0.000000000	4.202898000	0.659601000
C	0.000000000	-0.621156000	-4.212366000
C	0.000000000	-1.153979000	-2.893372000
C	0.000000000	-2.458458000	-2.463787000
C	0.000000000	-2.854065000	-1.137310000
C	0.000000000	-4.219763000	-0.698248000
C	0.000000000	-4.202898000	0.659601000
C	0.000000000	-2.827116000	1.072800000
C	0.000000000	-1.101656000	2.827942000
C	0.000000000	-0.679397000	4.202697000
C	0.000000000	0.679397000	4.202697000
C	0.000000000	1.101656000	2.827942000
C	0.000000000	2.415409000	2.393714000
C	0.000000000	-2.415409000	2.393714000
H	0.000000000	-1.348149000	5.049237000
H	0.000000000	1.348149000	5.049237000
H	0.000000000	5.040982000	1.339011000
H	0.000000000	5.073373000	-1.357883000
H	0.000000000	-5.073373000	-1.357883000
H	0.000000000	-5.040982000	1.339011000

H	0.000000000	3.228784000	-3.221759000
H	0.000000000	-3.228784000	-3.221759000
H	0.000000000	-3.189467000	3.149431000
H	0.000000000	3.189467000	3.149431000

7. Zn-porphyryne, C_{2v}, q = -1, S = ½, adiabatic anion

Zn	0.000000000	0.000000000	-0.045332000
N	0.000000000	2.062096000	-0.014461000
N	0.000000000	0.000000000	-2.051648000
N	0.000000000	-2.062096000	-0.014461000
N	0.000000000	0.000000000	2.026651000
C	0.000000000	4.244089000	-0.710484000
C	0.000000000	2.860919000	-1.125048000
C	0.000000000	1.094851000	-2.902537000
C	0.000000000	0.659200000	-4.294496000
C	0.000000000	2.411689000	-2.450886000
C	0.000000000	2.879627000	1.086164000
C	0.000000000	4.256101000	0.653925000
C	0.000000000	-0.659200000	-4.294496000
C	0.000000000	-1.094851000	-2.902537000
C	0.000000000	-2.411689000	-2.450886000
C	0.000000000	-2.860919000	-1.125048000
C	0.000000000	-4.244089000	-0.710484000
C	0.000000000	-4.256101000	0.653925000
C	0.000000000	-2.879627000	1.086164000
C	0.000000000	-1.105849000	2.841197000
C	0.000000000	-0.682879000	4.217433000
C	0.000000000	0.682879000	4.217433000
C	0.000000000	1.105849000	2.841197000
C	0.000000000	2.433524000	2.405505000
C	0.000000000	-2.433524000	2.405505000
H	0.000000000	-1.346871000	5.069498000
H	0.000000000	1.346871000	5.069498000
H	0.000000000	5.113262000	1.311518000
H	0.000000000	5.089239000	-1.382856000
H	0.000000000	-5.089239000	-1.382856000
H	0.000000000	-5.113262000	1.311518000
H	0.000000000	3.171124000	-3.223008000
H	0.000000000	-3.171124000	-3.223008000
H	0.000000000	-3.194420000	3.177437000
H	0.000000000	3.194420000	3.177437000

8. Zn-porphyrin, D_{4h}, q = 0, S = 0

Zn	0.000000000	0.000000000	0.000000000
C	0.000000000	3.428942000	0.000000000
C	0.000000000	-3.428942000	0.000000000
C	1.245822000	2.805210000	0.000000000
C	1.245822000	-2.805210000	0.000000000
C	2.520863000	3.482902000	0.000000000
C	2.520863000	-3.482902000	0.000000000
C	2.805210000	1.245822000	0.000000000
C	2.805210000	-1.245822000	0.000000000
C	3.428942000	0.000000000	0.000000000
C	3.482902000	2.520863000	0.000000000
C	3.482902000	-2.520863000	0.000000000
C	-1.245822000	2.805210000	0.000000000
C	-1.245822000	-2.805210000	0.000000000
C	-2.520863000	3.482902000	0.000000000
C	-2.520863000	-3.482902000	0.000000000
C	-2.805210000	1.245822000	0.000000000
C	-2.805210000	-1.245822000	0.000000000
C	-3.428942000	0.000000000	0.000000000
C	-3.482902000	2.520863000	0.000000000
C	-3.482902000	-2.520863000	0.000000000
H	0.000000000	4.511889000	0.000000000
H	0.000000000	-4.511889000	0.000000000
H	2.652278000	4.554194000	0.000000000
H	2.652278000	-4.554194000	0.000000000
H	4.511889000	0.000000000	0.000000000
H	4.554194000	2.652278000	0.000000000
H	4.554194000	-2.652278000	0.000000000
H	-2.652278000	4.554194000	0.000000000
H	-2.652278000	-4.554194000	0.000000000
H	-4.511889000	0.000000000	0.000000000
H	-4.554194000	2.652278000	0.000000000
H	-4.554194000	-2.652278000	0.000000000
N	1.449762000	1.449762000	0.000000000
N	1.449762000	-1.449762000	0.000000000
N	-1.449762000	1.449762000	0.000000000
N	-1.449762000	-1.449762000	0.000000000

9. Zn-porphyrin, C_1, q = 0, S = 1

Zn	0.013267000	-0.008818000	-0.000001000
C	0.004348000	3.426374000	-0.000001000
C	0.020538000	-3.443660000	-0.000004000
C	1.269828000	-2.822830000	-0.000003000
C	1.270407000	2.807922000	-0.000002000
C	2.521922000	3.476337000	-0.000001000
C	2.546039000	-3.507550000	-0.000002000
C	2.822378000	1.249558000	0.000000000
C	2.839316000	-1.269697000	-0.000001000
C	3.451939000	-0.038276000	0.000001000

C	3.499102000	2.493527000	0.000000000
C	3.510398000	-2.552677000	0.000002000
C	-1.244913000	2.805011000	0.000001000
C	-1.245545000	-2.825237000	-0.000001000
C	-2.496996000	-3.493976000	0.000002000
C	-2.521210000	3.489882000	0.000001000
C	-2.797864000	-1.267253000	0.000000000
C	-2.814405000	1.251998000	0.000000000
C	-3.427200000	0.020707000	-0.000001000
C	-3.474336000	-2.511500000	0.000002000
C	-3.485451000	2.534996000	0.000001000
H	0.002189000	4.509796000	-0.000003000
H	0.022372000	-4.527093000	-0.000004000
H	2.658154000	4.546694000	0.000000000
H	2.672185000	-4.579482000	-0.000002000
H	4.535339000	-0.033776000	0.000003000
H	4.570051000	2.623982000	0.000003000
H	4.581012000	-2.689443000	0.000006000
H	-2.633305000	-4.564322000	0.000003000
H	-2.647192000	4.561816000	0.000002000
H	-4.510617000	0.016462000	-0.000002000
H	-4.545251000	-2.642349000	0.000003000
H	-4.556080000	2.671439000	0.000001000
N	1.470891000	1.442371000	-0.000002000
N	1.471980000	-1.473678000	-0.000002000
N	-1.446447000	-1.459909000	-0.000001000
N	-1.446711000	1.456106000	0.000000000

10. Zn-porphyrin, C_{4h}, q = +1, S = ½, adiabatic cationic state 1

Zn	0.000000000	0.000000000	0.000000000
C	0.093521000	-4.309341000	0.000000000
C	0.578337000	3.021640000	0.000000000
C	1.424927000	-4.059461000	0.000000000
C	1.580629000	-2.616687000	0.000000000
C	1.939366000	2.825713000	0.000000000
C	2.616689000	1.580616000	0.000000000
C	2.825732000	-1.939370000	0.000000000
C	3.021663000	-0.578349000	0.000000000
C	4.059461000	1.424912000	0.000000000
C	4.309363000	0.093509000	0.000000000
C	-0.093521000	4.309341000	0.000000000
C	-0.578337000	-3.021640000	0.000000000
C	-1.424927000	4.059461000	0.000000000
C	-1.580629000	2.616687000	0.000000000
C	-1.939366000	-2.825713000	0.000000000
C	-2.616689000	-1.580616000	0.000000000
C	-2.825732000	1.939370000	0.000000000
C	-3.021663000	0.578349000	0.000000000
C	-4.059461000	-1.424912000	0.000000000

C	-4.309363000	-0.093509000	0.000000000
H	0.404835000	5.266207000	0.000000000
H	2.236126000	-4.770729000	0.000000000
H	2.562956000	3.710648000	0.000000000
H	3.710680000	-2.562943000	0.000000000
H	4.770696000	2.236139000	0.000000000
H	5.266241000	-0.404829000	0.000000000
H	-0.404835000	-5.266207000	0.000000000
H	-2.236126000	4.770729000	0.000000000
H	-2.562956000	-3.710648000	0.000000000
H	-3.710680000	2.562943000	0.000000000
H	-4.770696000	-2.236139000	0.000000000
H	-5.266241000	0.404829000	0.000000000
N	0.371707000	-2.013572000	0.000000000
N	2.013595000	0.371692000	0.000000000
N	-0.371707000	2.013572000	0.000000000
N	-2.013595000	-0.371692000	0.000000000

11. Zn-porphyrin, D_{2h}, q = -1, S = ½, adiabatic anion

Zn	0.000000000	0.000000000	0.000000000
C	0.000000000	3.437354000	0.000000000
C	0.000000000	-3.437354000	0.000000000
C	1.249843000	2.823093000	0.000000000
C	1.249843000	-2.823093000	0.000000000
C	2.514579000	3.494228000	0.000000000
C	2.514579000	-3.494228000	0.000000000
C	2.815475000	1.251438000	0.000000000
C	2.815475000	-1.251438000	0.000000000
C	3.448903000	0.000000000	0.000000000
C	3.486635000	2.528033000	0.000000000
C	3.486635000	-2.528033000	0.000000000
C	-1.249843000	2.823093000	0.000000000
C	-1.249843000	-2.823093000	0.000000000
C	-2.514579000	3.494228000	0.000000000
C	-2.514579000	-3.494228000	0.000000000
C	-2.815475000	1.251438000	0.000000000
C	-2.815475000	-1.251438000	0.000000000
C	-3.448903000	0.000000000	0.000000000
C	-3.486635000	2.528033000	0.000000000
C	-3.486635000	-2.528033000	0.000000000
H	0.000000000	4.522202000	0.000000000
H	0.000000000	-4.522202000	0.000000000
H	2.645161000	4.567039000	0.000000000
H	2.645161000	-4.567039000	0.000000000
H	4.532165000	0.000000000	0.000000000
H	4.558482000	2.663934000	0.000000000
H	4.558482000	-2.663934000	0.000000000
H	-2.645161000	4.567039000	0.000000000
H	-2.645161000	-4.567039000	0.000000000

H	-4.532165000	0.000000000	0.000000000
H	-4.558482000	2.663934000	0.000000000
H	-4.558482000	-2.663934000	0.000000000
N	1.462082000	1.452823000	0.000000000
N	1.462082000	-1.452823000	0.000000000
N	-1.462082000	1.452823000	0.000000000
N	-1.462082000	-1.452823000	0.000000000
